# Rhabdomyolysis in a Child Secondary to *Staphylococcus aureus* Endocarditis

**DOI:** 10.4103/0974-777X.56250

**Published:** 2009

**Authors:** Srinivas Bandi, Ashish Chikermane

**Affiliations:** *Department of Paediatrics, Birmingham Children's Hospital, Steelhouse Lane, Birmingham, B4 6NH, UK*; 1 Department of Paediatric Cardiology, Birmingham Children's Hospital, Steelhouse Lane, Birmingham, B4 6NH, UK

**Keywords:** Endocarditis, Rhabdomyolysis, *Staphylococcus aureus*

## Abstract

Rhabdomyolysis secondary to bacterial infection has only rarely been investigated, and there are case reports of the same mainly in adults. This article describes the first reported case of rhabdomyolysis in a child secondary to *Staphylococcus aureus* endocarditis. A 12-year-old child presented with myalgia, pyrexia and dark urine and was found to have infective endocarditis due to *S. aureus.*

## INTRODUCTION

Rhabdomyolysis is the breakdown of muscle fibers, when leakage of potentially toxic cellular contents into the circulation can lead to hypovolemia, acidosis, hyperkalemia, acute renal failure and disseminated intravascular coagulation.[1] An increased plasma concentration of creatine kinase (CK) or myoglobin and the presence of myoglobinuria are useful parameters for diagnosing this disease. Alcohol ingestion, crush injuries and generalised seizures are well-known causes of rhabdomyolysis.[1] Rhabdomyolysis secondary to bacterial infection has only rarely been investigated, and there are case reports of the same mainly in adults.[2–11]

This article describes the first reported case of rhabdomyolysis in a child secondary to *Staphylococcus aureus* endocarditis. A 12-year-old child presented with myalgia, pyrexia and dark urine and was found to have infective endocarditis due to *S. aureus.*

## CASE REPORT

A 12-year-old boy was admitted with a 1-week history of pyrexia, myalgia and dark urine. He had history of herpes encephalitis and epilepsy and was not on any other medication apart from carbamazepine for his epilepsy. He did not have any seizures recently, and there was no history of trauma. There was no previous history of muscle disease or muscle weakness. He was completely mobile till the occurrence of this current illness. On examination he was febrile, with a normal capillary refill and blood pressure. He had a few infected blisters on his legs. There were no signs and symptoms suggestive of alcohol intoxication. There was generalized tenderness over his muscles, and he could not bear weight without support because of myalgia. Findings from his neurological examination were completely normal, including tendon reflexes and fundoscopy. He had normal heart sounds with no murmurs, his chest was clear, his abdomen was soft and there was no hepatosplenomegaly.

His urine dipstick showed 3+ blood, and subsequent laboratory testing confirmed it as myoglobin. He had a raised C-reactive protein (CRP) of 225 U/L and a raised creatine kinase of 9982 U/L. He was initially started on cefotaxime, which was changed to more specific flucloxacillin and rifampicin once the initial blood culture grew S*taphylococcus aureus* (sensitive to flucloxacillin). His creatine kinase reached a peak value of 12156 U/L on day 2 of admission, with a gradual fall over the next few days, reaching a normal level by day 7 of admission. The myalgia improved gradually, he started bearing weight after 2 to 3 days and was completely mobile by day 5 of admission. He remained hemodynamically stable throughout his hospital stay without any evidence of shock. His renal functions were normal, apart from slightly raised urea, viz., 9.4, which settled with adequate intravenous hydration.

On day 4 of admission, he developed a heart murmur; and in view of persisting pyrexia, he underwent an echocardiogram, which showed large vegetation on the septal leaflet of tricuspid valve. Gentamicin was added; however, even after 2½ weeks of intravenous antibiotic therapy, he continued to have high-grade fever. Both the fever and the inflammatory markers settled once the vegetation was surgically removed.

He received antibiotics for a total 6 weeks (4 weeks IV and 2 weeks oral). The vegetation did not grow any bacteria, and the histology was consistent with infective endocarditis. The follow-up echocardiograms have all been normal.

## DISCUSSION

The pathogenesis of rhabdomyolysis is not fully understood. However, direct bacterial invasion into muscle,[12] bacterial toxin,[13] metabolic derangements,[13] rigors and fever[14] and nonspecific sepsis-related mechanisms[12,15,16] may be precipitating factors that explain muscle injury. Although there is no supporting evidence, it has been suspected that endotoxin or endotoxin-like substances might play a part in the onset of rhabdomyolysis.[6]

In our case, there was no other contributing history to suggest other causes of rhabdomyolysis — there was no history of trauma; he did not have recent seizures; he was not on any known medication that could potentially cause rhabdomyolysis. There was definite clinical (myalgia, muscle tenderness) and biochemical (raised CK) evidence of muscle inflammation. His inability to bear weight was attributed to myalgia as he had normal tendon reflexes and muscle power. Hence a neurology consultation or neuroimaging was not requested. The only contributing factor for rhabdomyolysis in our case was staphylococcal sepsis. His symptoms and signs of rhabdomyolysis improved within 1 week, although he developed further complication of bacterial endocarditis, which required prolonged treatment.

Muscle involvement by *S. aureus* usually manifests as suppurative myositis — either as a localized large abscess or many small abscesses. This condition has been named “pyomyositis.”[17] Rhabdomyolysis has also been noted in patients with toxic shock syndrome,[18–21] presumably secondary to the effects of exotoxin on the muscle.

Two previous cases of rhabdomyolysis associated with *S. aureus* have been reported in children without toxic shock. Adamski and co-workers described the case of a 15-year-old boy with nonsuppurative myositis and rhabdomyolysis.[8] The creatine kinase level was 33,400 U/L. Saul and co-workers reported a case of a 13-year-old girl with rhabdomyolysis with a creatine kinase level of 59,729 U/L. She did not have toxic shock syndrome or bacteremia but had staphylococcus organisms isolated from urine.[22]

To the best of our knowledge, there is only one previous case report of rhabdomyolysis in a 20-year-old man who had *S. aureus* endocarditis.[10] In that case, blood and urine cultures were positive for *S. aureus,* and the peak creatine kinase level was 990 U/L. To date there are no similar reports in pediatric literature. In our case the blood culture was positive for *S. aureus,* and echocardiogram demonstrated vegetation, confirming the diagnosis of infective endocarditis. This is probably the most feasible explanation for the child's symptoms. The infected blisters were probably the potential source of his infection, though we haven't grown anything from the blisters. In spite of initial antibiotic treatment, the fever persisted and only settled once the vegetation was surgically removed. Luckily he had only mild derangement of his renal functions but could have potentially had a full-blown renal failure requiring intensive management.

It has been reported that about 33% of patients with rhabdomyolysis develop acute renal failure.[23] However, it is also recognized that myoglobinuria itself does not cause acute renal failure, and the renal dysfunction seems to result from a combination of hypovolemia, hypoxia, endotoxin- or endotoxin-like substances with other events.

Once the diagnosis of rhabdomyolysis is made, adequate hydration and alkalinization of the urine are necessary in order to prevent renal failure. In our patient suitable antibiotic treatment and adequate hydration proved effective in slowing the process of rhabdomyolysis.

## CONCLUSION

We conclude that rhabdomyolysis should be considered as a possible complication in various infections, especially in any child presenting with myalgia and dark urine. Early aggressive management has a good prognosis.
